# Effect of Natural Preservatives on the Nutritional Profile, Chemical Composition, Bioactivity and Stability of a Nutraceutical Preparation of *Aloe arborescens*

**DOI:** 10.3390/antiox9040281

**Published:** 2020-03-26

**Authors:** Filipa A. Fernandes, Márcio Carocho, Sandrina A. Heleno, Paula Rodrigues, Maria Inês Dias, José Pinela, Miguel A. Prieto, Jesus Simal-Gandara, Lillian Barros, Isabel C. F. R. Ferreira

**Affiliations:** 1 Centro de Investigação de Montanha (CIMO), Instituto Politécnico de Bragança, Campus de Santa Apolónia, 5300-253 Bragança, Portugal; filipaf@ipb.pt (F.A.F.); sheleno@ipb.pt (S.A.H.); prodrigues@ipb.pt (P.R.); maria.ines@ipb.pt (M.I.D.); jpinela@ipb.pt (J.P.); iferreira@ipb.pt (I.C.F.R.F.); 2 Nutrition and Bromatology Group, Department of Analytical and Food Chemistry, Faculty of Food Science and Technology, University of Vigo-Ourense Campus, E-32004 Ourense, Spain; mprieto@uvigo.es (M.A.P.); jsimal@uvigo.es (J.S.-G.)

**Keywords:** nutraceuticals, natural ingredients, *Aloe arborescens*, natural preservatives, chestnut flowers

## Abstract

Citric acid, quercetin, dried chestnut flowers and an aqueous extract of chestnut flowers were screened as candidates for preserving a drinkable nutraceutical preparation for 45 days. The assays encompassed antioxidant and antimicrobial activities, nutritional and chemical profiles, and individual profiles of fatty acids and mineral composition, all of which in comparison with a sodium benzoate, a synthetic preservative. The centesimal composition of the nutraceutical formulation was mainly composed of carbohydrates, followed by proteins and fat, with moisture levels between 66% and 71%. Palmitic and stearic acid were the most abundant fatty acids, while calcium and magnesium where the minerals in higher amount. Anthroquinones, followed by flavonoids where the most abundant groups of phenolic compounds. In terms of the preserving effects of the extracts, the chestnut flowers and the citric acid were the most effective natural preservatives, which better preserved phenolic compounds. Furthermore, these two ingredients also revealed the strongest capacity to control the microbial growth in the formulation by inhibiting the growth of food contaminants. In general, these ingredients revealed higher preservation capacity than sodium benzoate, while not altering the nutritional and fatty acid profile. The chestnut flowers and citric acid could be used to preserve foods, food supplements, and nutraceutical formulations after passing the required regulatory procedures for food additives.

## 1. Introduction

Nutraceutical is a recent term which does not have a formal regulatory definition; it is a combination of the word nutrient and pharmaceutical. It was first defined by Stephen DeFelice as “food or part of a food that provides medical or health benefits, including the prevention and/or treatment of a disease”, therefore, this concept is often confused with the term food supplement as well as functional food. However, a legally defined food supplement is a food product with nutritional or physiological effects for the purpose of diet supplementation and is marketed in dosage formulas. A functional food is considered a conventional food product that provides consumer health benefits which can be altered by adding or removing compounds to result in a food rich in specific bioactive molecules [1,2,3].

The medicinal properties of plants have been well known from an early age, and while knowledge begun empirically, today there are many scientific studies supporting these claims. *Aloe* is a genus known for its medicinal properties, comprising over 500 species [4]. There are several studies, in vitro and in vivo, that prove its pharmacological properties, including, antioxidant, antimicrobial and anti-inflammatory activity, among others [5,6]. *Aloe Arborescens* Mill., is one of the most abundant species of the *Aloe* genus, native to South Africa which has been imported into many countries as an ornamental and medicinal plant due to its biological effects, namely antimicrobial, anti-inflammatory, and scab healing properties, which have been proven by scientific studies [7]. Beyond this, *A. arborescens* is used for extraction of active ingredients with cosmetic and nutraceutical interest [8].

There is a whole new array of foods and products that have been developed to provide health benefits to consumers. Due to the fast-paced lives of individuals, these nutraceuticals have been gaining considerable interest. Their top benefits are, beyond delivering nutritional value, being functional and offering high amounts of beneficial compounds, mostly of natural origin, with low calories. The growing concern about the amount of chemicals added to food as well as pharmaceutical and cosmetic products have tilted consumer preference towards products with few additives, and, when available, preference for natural additives, which are perceived by consumers as less harmful to human health [9,10]. Sodium benzoate, an artificial preservative widely used in the food industry, has been linked to nefarious effects to the human health, especially in long term exposure [11]. Bruna et al. (2018) [12] reviewed sodium benzoate as a preservative and concluded that when consumed above the recommended daily intake (5 mg/kg), achieved due to its high usage in different foods, may have adverse health effects for consumers. In order to adapt to consumer preference, industries are looking for innovative and inexpensive natural solutions, so the demand for these additives has increased significantly [13]. Concerning preservative additives (substances that retard or prevent the deterioration of products from microorganisms or oxidation), there is a great advance in the use of natural matrices, and several studies have shown the effectiveness of these compounds in the preservation of products [14,15,16]. Carocho et al. studied the preservative capacity of chestnut flowers when incorporated into “Serra da Estrela” cheese, and found that they capable of preserving this cheese [13].

In order to find a natural alternative for sodium benzoate (E211) as a preservative for a drinkable nutraceutical preparation made from *A. arborescens*, various alternatives were tested. These natural alternatives should be effective during the whole span of the nutraceutical’s shelf-life after opening, which was set at 30 days. Still, the nutraceutical was analyzed for 45 days in order to attain the possibility of a shelf-life extension. To achieve concrete results, various dimensions of the nutraceutical preparations were analyzed, namely the centesimal and chemical composition, antioxidant and antimicrobial capacity, as well as individual fatty acid and phenolic compounds.

## 2. Materials and Methods

### 2.1. Sample Preparation

The *A. arborescens* samples were provided by the Aloeforlife enterprise (Oeiras, Portugal), as well as the other two ingredients needed for the nutraceutical preparation; honey, and fig brandy. Upon reception of the samples, the preparation of the nutraceutical formulation was carried out: the leaves of *A. arborescens* were disinfected for 20 min with sodium dichloroisocyanurate (a commonly used food disinfectant) prior to the removal of the spikes. The leaves were then cut into small segments of about 2 cm. Next, 315 g of sliced leaves were added to 175 g of honey and 10 mL of fig brandy, and blended to a pulp for 15 min. Finally, the nutraceutical preparation was stored in 500 mL amber flasks. Six lots of three flasks each were prepared, one for each of the tested preservatives. Lot 1 was the negative control group, without any preservative; lot 2 was the positive control, containing 1 g of sodium benzoate; lot 3 was preserved with 1 g of citric acid, maintaining the same amount as the positive control; lot 4 used 1 g of quercetin, lot 5 was preserved with dry milled chestnut flower (5.3 g), and finally lot 6 was preserved with an extract of chestnut flower which was obtained by infusion and added at 47 mL for the 500 mL of nutraceutical [13]. The final step consisted of a blending for five minutes with three repetitions, to reduce the ingredients to a liquid pulp. All flasks were sealed prior to being opened for the T0 sample collection, and kept closed, away from light sources for the remainder of the assay. After 30 and 45 days, samples were collected from all flasks for analysis. Before analysis, the samples were lyophilized (FreeZone 4.5, Labconco, Kansas City, MO, USA) and stored at −24 °C.

### 2.2. Standards and Reagents

All reagents were acquired from scientific retailers, and were, at lest of P.A. purity, except when used for High Performance Liquid Chromatography (HPLC), which were of HPLC grade.

### 2.3. Antioxidant Activity of the Nutraceutical Formulations

The antioxidant activity was measured through two different assays, namely the thiobarbituric acid reactive substances inhibition (TBARS) and the oxidative hemolysis inhibition assays (OxHLIA). Trolox was used as positive control for both assays.

#### 2.3.1. Thiobarbituric Acid Reactive Substances (TBARS) Analysis

The lyophilized extracts were re-dissolved in water at a concentration of 40 mg/mL. The stock solutions were subjected to successive dilutions for analysis, ranging from 0.04 to 40 mg/mL. The antioxidant capacity was measured by the decrease of TBARS using porcine (*Sus scrofa*) brain homogenates. The color intensity of the malondialdehyde–thiobarbituric acid (MDA–TBA) (Thermo Fisher Scientific Co., Waltham, MS, USA) complex in the supernatant was measured by its absorbance at 532 nm, following a procedure described previously by Sarmento et al. [17]. The results were expressed in EC_50_ value (mg/mL extract).

#### 2.3.2. Oxidative Hemolysis Inhibition Assay

The OxHLIA assay followed a procedure described previously by Mandim et al. [18] in which a known weight of extract was dissolved in phosphate buffered saline solution (PBS) (Thermo Fisher Scientific Co., Waltham, MS, USA), obtaining different concentrations ranging from 10 µg mL^−1^ to 25 mg mL^−1^. To determine the inhibition capacity of the oxidative hemolysis associated with each of the tested extracts, blood from healthy sheep was harvested and the isolated erythrocytes were used as substrate. Ethical animal measures were used in terms of the blood harvesting. Small amounts of blood were harvested by the Veterinarian of the Polytechnic Institute of Bragança from the institution’s herd, guaranteeing no suffering towards the animals. Collection was carried out in random animals, with jugular extraction of the blood using a proper syringe. The blood was then pooled and brought to laboratory for further analysis. The prepared erythrocyte solution (2.8%, *v*/*v*) was mixed with either extract solutions, PBS (control), or water (for complete hemolysis). After pre-incubation at 37 °C for 10 min with shaking, 2,2’-Azobis(2-amidinopropane) dihydrochloride (Thermo Fisher Scientific Co., Waltham, MS, USA) (160 mM was added, and the optical density was measured at 690 nm every 10 min until complete hemolysis. The extract concentration required to keep 50% of the erythrocyte population intact for a Δ*t* (time interval) of 60 to 120 min was calculated and expressed as IC50 values (µg/mL) for both Δ*t*.

### 2.4. Nutritional Profile of the Nutraceutical Formulations

The different nutraceutical preparations were analyzed in terms of their contents in moisture, energy and macronutrients (fat, ash, proteins, and carbohydrates) according to AOAC (Association of Official Agricultural Chemists) procedures for the three time periods, T0, immediately after preparation, 30 days after opening, and finally after 45 days. Moisture was determined following the AOAC 925.09 method, crude protein (N×6.25) was determined by the macro-Kjeldahl method (AOAC 920.87); the crude fat was determined using a Soxhlet apparatus by extracting a known weight of powdered sample with petroleum ether (AOAC 989.05); ash was estimated by incineration at 600 ± 15 °C for 5 h (AOAC 923.03). Total carbohydrates were calculated by difference and the energy was estimated using the following equation: Energy (kcal) = 4 × (g protein + g carbohydrates) + 9 × (g fat). The results were expressed in g/100g of fresh weight (fw) and both in Kcal and KJ for energy values [19].

### 2.5. Fatty Acid Profiles

Fatty acids were determined by Gas Chromatography (GC) coupled with a flame ionization detector (DANI model GC 1000, Contone, Switzerland). The separation was achieved with a Macherey–Nagel (Düren, Germany) column (50% cyanopropyl-methyl–50% phenylmethylpolysiloxane, 30 m × 0.32 mm i.d. × 0.25 μm film thickness). Fatty acids were identified by comparing their retention times to the ones of FAME (fatty acids methyl esters) peaks from samples with commercial standards (FAME reference standard mixture, standard 47885-U, Sigma-Aldrich, St. Louis, MO, USA). The results were recorded and processed using the CSW 1.7 Software (DataApex 1.7, Prague, Czech Republic) and were expressed in relative percentage (%).

### 2.6. Mineral and Chemical Composition of the Nutraceutical Formulations

#### 2.6.1. Mineral Profiles

Minerals were determined by atomic absorption spectrophotometry (AAS), in which potassium (K), sodium (Na), calcium (Ca), and magnesium (Mg) were detected using a Pye Unicam PU9100X spectrophotometer (Cambridge, United Kingdom), and manganese (Mn), zinc (Zn), iron (Fe), and copper (Cu) were detected using a Perkin Elmer PinAAcle 900 spectrophotometer (Waltham, MA, USA). For the determination, 1 g of sample was weighed and digested with 10 mL of nitric acid, using heat and microwaves, at 200 °C and 1200 W for 15 min. After cooling down, the samples were analyzed by AAS, with prior treatment for specific elements. For the determination of potassium and sodium the sample was diluted in a caesium buffer (Thermo Fisher Scientific Co., Waltham, MS, USA) (1:10 mL), for calcium and magnesium the sample was diluted in a lanthanum solution (Thermo Fisher Scientific Co., Waltham, MS, USA) (10 g/L) and for manganese and copper a magnesium nitrate solution was used as a matrix modifier. The determination of elements was achieved by comparing the absorbance responses to pure analytical solutions. The results were expressed in mg/100 g fw.

#### 2.6.2. Free Sugars and Organic Acids

The chemical composition, encompassing free sugars and organic acids was evaluated following procedures previously described by Barros et al. [20].

Free sugars were analyzed by HPLC coupled to a refraction index detector (Knauer, Smartline system 1000). The compounds were identified by chromatographic comparisons with authentic standards (D(−)-fructose, D(+)-sucrose, D(+)-glucose, D(+)-trehalose, and D(+)-raffinose pentahydrate) which were purchased at Sigma-Aldrich (St. Louis, MO, USA), as also melezitose (PanReac AppliChem ITW Reagents Co., Darmstadt, Germany) which was applied as the internal standard (IS) and used in the quantification method. Data was analyzed using Clarity 2.4 software (DataApex, Podohradska, Czech Republic), and the results were expressed in g/100 g fw.

Organic acids were evaluated using an Ultra-Fast Liquid Chromatography (UFLC, Shimadzu 20A series, Kyoto, Japan) coupled to a diode array detector. Organic acids standards (L(+)-ascorbic acid, citric acid, malic acid, oxalic acid, shikinic acid, succinic acid, fumaric acid, and quinic acid; Sigma-Aldrich, St. Louis, MO, USA) were used for identification by performing chromatographic comparisons with the peaks of the samples. These standards were also used for quantification relying on the external standard methodology. Results were expressed g/100 g fw.

### 2.7. Phenolic Compounds

The phenolic compounds present in the nutraceutical preparations were determined following the procedures published by Pinela et al. [21]. The different nutraceutical formulations were extracted by maceration with ethanol/water (80:20 *v/v*; 30 mL), during 1 h at room temperature, being the supernatant filtered (Whatman No. 4 paper), and the residue re-extracted with the same extraction solvent. After evaporating the ethanol at 40 °C (rotary evaporator Büchi R-210, Flawil, Switzerland) the aqueous phase of the extract was lyophilized (FreeZone 4.5, Labconco, Kansas City, MO, USA).

The lyophilized extracts were purified using Sep-Pak C18 3 cc Vac Cartridges (Phenomenex, Torrance, CA, USA) activated with methanol and water. The samples (at ~20 mg/mL in water; 2.5 mL) were eluted through the cartridge, while sugars and other polar substances were removed by passing 5 mL of water. The phenolic compounds were further eluted with methanol (5 mL), and the methanol was evaporated at 40 °C. Thereafter, the purified extracts were re-dissolved in ethanol/water (20:80, *v/v*; 1 mL), and filtered through 0.22-μm disposable Liquid Chromatography filter disks. The chromatographic analysis was achieved by using a Dionex Ultimate 3000 UPLC (Thermo Scientific, San Jose, CA, USA), coupled to a diode array detector (280, 330, and 370 nm) and an electrospray ionization mass detector (Linear Ion Trap LTQ XL, Thermo Finnigan, San Jose, CA, USA), working in negative mode. The chromatographic separation was performed using a Waters Spherisorb S3 ODS-2 C18 (3 μm, 4.6 mm × 150 mm, Waters, Milford, MA, USA) column at 35 °C. The compounds were identified considering their retention time, UV-Vis and mass spectra in comparison with available standards and literature data. Calibration curves of the available phenolic standards were plotted based on the UV-Vis signal in order to obtain quantitative analysis. In the case of unavailable commercial standards, the compounds were quantified via calibration curve of the most similar standard available. The results were expressed as mg/g of extract.

### 2.8. Microbiological Analysis

#### 2.8.1. Contamination Procedure

In order to understand the effectiveness of the natural preservatives against food contaminants, portions of the nutraceutical preparations were contaminated with food pathogens, and the development or subsiding of the colonies was evaluated. All nutraceutical formulations were contaminated with *Escherichia coli*, *Bacillus cereus*, *Aspergillus parasiticus*, and *Zygosaccharomyces rouxii*, kindly provided by the Mountain Research Centre of the Polytechnic Institute of Bragança (Bragança, Portugal) and were selected due to their prevalence in this kind of foods (plant or plant derivatives). For the preparation of the pre-inocula, bacterial cells from 1-day-old cultures were suspended in Tryptic Soy Broth (TSB, Liofilchem Co., Roseto degli Abruzzi, Italy) and incubated at 36 °C for 24 h. The yeast was suspended in TSB medium and incubated at 25 °C for 24 h. Optical density of the suspensions was determined using a densitometer Den-1B (Biosan, Riga, Latvia). Cell concentration was estimated to be equal to 10^9^ cells/mL. The fungal pre-inoculum was prepared by inoculation in Malt Extract Agar (MEA, Liofilchem Co., Roseto degli Abruzzi, Italy) and incubation for 7 days at 25 °C in the dark. After incubation, 2 mL of 0.05% Tween 80 solution were added to the culture and spores were scrubbed to obtain a suspension. Spore suspension was adjusted to 10^9^ spores/mL with the aid of a Neubauer counting chamber. All samples were contaminated with approximately 10^6^ cell/mL of each microorganism, and the number of viable cells (in CFU/mL) was confirmed by the plate count technique.

#### 2.8.2. General Sample Preparation

The preparation of samples for the microbial load analysis followed the procedure described in the International Organization for Standardization [22]. The different nutraceutical preparations (5 mL) were mixed with 45 mL of peptone water in stomacher bags and further homogenized in a stomacher equipment (Star Blender, VWR, Radnor, USA) for 1 min at 300 units. The obtained suspensions were further diluted to obtain dilutions from 10-1 to 10-5. Each solution was analyzed in duplicate.

#### 2.8.3. Microorganism Analysis

*Coliforms (and E. coli; ISO 4832:2006)*: The dilutions were inoculated in VRBLA (violet red bile lactose agar) (Liofilchem Co., Roseto degli Abruzzi, Italy) by the pour plate technique, in duplicate (Limit of Quantification (LOQ) = 1 log (Conoly-Forming Units) CFU/g): 1 mL of suspension was pipetted into the plate and 15 mL of melted VRBLA (Liofilchem Co., Roseto degli Abruzzi, Italy) (kept at 50 °C in a water bath or incubator) were poured prior to homogenization, and left to solidify. On the top of the medium, a layer of 4 mL of VRBLA was poured and was left to solidify. The plates were incubated at 30 °C for 48 h, in reversed position. Counting was performed in plates that had between 10 and 150 colonies.

*Yeasts and Moulds (ISO 21527-1/2:2008)*: The dilutions were inoculated in DRBC (dichloran rose bengal chloramphenicol) (Liofilchem Co., Roseto degli Abruzzi, Italy) by the spread plate technique, in duplicate (LOQ = 1.7 log CFU/g): 0.2 mL of the suspensions were pipetted onto a plate containing 15 mL of the medium and were spread with a disposable spreader. Incubation was set at 25 °C for 5 days, in the upright position. In the plates having less than 150 colonies, the count of yeast and mold colonies was performed separately after 2 and 5 days of incubation.

*Bacillus cereus (ISO 7932:2004)*: The dilutions were inoculated in MYP (mannitol yolk polymyxin) (Liofilchem Co., Roseto degli Abruzzi, Italy) by the spread plate technique, in duplicate (LOQ = 1.7 log CFU/g): 0.2 mL of suspension were pipetted onto a plate containing 15 mL of the medium and were spread with a disposable spreader. Incubation was performed at 30 °C for 24–48 h, in reversed position. Counting was performed in the plates having between 10 and 150 colonies. All counts of the microbial load were performed at the same intervals as all other assays of this work, namely T0—immediately after preparation and contamination, T30—after 30 days which was the shelf-life of the nutraceutical after opening, and T45, which was beyond the shelf-life.

### 2.9. Statistical Analysis

Throughout the manuscript, all values are represented as mean ± standard deviation (SD), and all analysis are carried out using a significance level of 0.05. In order to aid the interpretation of the results, the analytical profiles of the different nutraceutical formulations were treated through a 2-way analysis of variance (ANOVA). This type of statistical analysis helps increase the clarity of results by allowing the quantification (when possible) of each contributing factor individually. In this manuscript, there are two-factors that contribute to changes in the analytical profiles, the storage time (ST), and the type of preservative (TP). If a significant interaction is found among the two factors (*p* < 0.05 TP×ST) they were simultaneously evaluated, and some general conclusions were, when available, drawn from the Estimated Marginal Mean Plots (EMM). If no significant interaction was detected (*p* > 0.05), each factor was evaluated simultaneously and classified through a Tukey’s HSD test for homoscedastic samples and a Tamhane’s T2 test for non-homoscedastic samples. Homoscedasticity was evaluated using a Levene’s test. In addition, a linear discriminant analysis (LDA) was employed to compare the different preservatives as well as to determine the main differences occurring in the samples throughout the 45 days of storage. The LDA used the Wilk’s λ test with an *F*-value of 3.84 for entering and 2.71 for removal. For the model performance, a leave-one-out cross validation procedure was used.

## 3. Results and Discussion

The main objective of this work was to find natural alternatives to sodium benzoate, an artificial yet authorized food additive within the European Union, for a commercial preparation of a vegetable nutraceutical. The candidates had to show antioxidant and antimicrobial activity for at least 30 days (the shelf-life of the nutraceutical after opening). Furthermore, the preservatives should not induce a deep impact on the nutritional and chemical profiles of the formulation, in line with the definition of food additive for the European Food Safety Authority (EFSA) [10]. For this, several options were assessed, namely extracts, pure natural compounds obtained from plants, and synthetic compounds with natural origin. A total of four different alternatives were tested, namely citric acid (an organic acid), quercetin (a flavonoid), dry milled chestnut flowers, and the aqueous extract of chestnut flower obtained by infusion. To the best of the author’s knowledge, this is a first report on the use of natural extracts and compounds as natural preservatives in nutraceuticals. This novelty should raise the interest of the scientific community and industry to pursuit other natural alternatives to preserve nutraceuticals.

### 3.1. Antioxidant and Nutritional Profile

Table 1 is divided into two sections, an upper one with the different types of preservatives analyzed (TP) and the lower one with the three storage times (ST) (T0, T30, and T45). As stated in Section 2.7., the representation of values in this manner, a 2-way ANOVA, allows the determination of the interaction of both factors, TP and ST. Thus, each different preservative formulation comprises all storage times, and each storage time of the lower section comprises all preservative formulations. The antioxidant capacity of the different formulations is shown in Table 1, on the left the TBARS and two OxHLIA assays, at 60 and 120 min.

Regarding the TBARS assay, this analysis was carried out to verify the capacity of the preservatives to inhibit the formation of thiobarbituric and malodialdehyde complexes, produced during lipid peroxidation as secondary products [23]. Considering TP×ST, there was no significant interaction, therefore, individual classifications were extracted, and, regarding the preservative type, there were no significant differences. The best preserving capacity was found for quercetin and chestnut flowers preserved formulations, which showed the lowest EC_50_, followed by the chestnut extract preserved formulation. The control sample scored better than the citric acid and sodium benzoate. Still, sodium benzoate also proved to not have a very interesting preserving capacity in terms of lipid protection. Regarding the three different storage times, there was no significative difference among them.

OxHLIA assays, presented as IC_50_, represent the extract concentration needed to protect 50% of the erythrocyte population from the oxidative hemolysis caused by the thermal decomposition of AAPH for a Δ*t* of 60 and 120 min. The lower the IC_50_ value the higher the antioxidant activity of the extract. The IC_50_ were calculated at two Δ*t* because natural extracts contain different antioxidants which can interact with each other and offer protection at different times. There was a significant interaction (TP×ST < 0.05), and thus both the storage time, and the preservative type had an influence on the outcome, not allowing general concrete conclusions. Still, comparing the 60- to the 120-min assay, there seems to be lower values for the 60 min, showing that all the extracts have higher activity at an initial phase. Still, due to a significative interaction among the storage times and the different preservatives used, no classifications could be carried out.

Table 1 also shows the nutritional profile of the different nutraceutical formulations (right of the vertical dashed line), revealing that the most abundant nutrients are the carbohydrates, with values between 98 and 99 g/100 g of fresh weight. Proteins are the second most prevalent nutrients, although all registered below 1g/100 g. All nutraceutical preparations showed moisture ranging between 66 and 71%, which was expected, provided that the main ingredient of the nutraceutical preparation, *A. arborescens* leaves, are about 99% water [24], and represent about 66% of the whole formulation, whilst honey composes the other 33%. In terms of classification, there were significative interactions between ST and TP for fat, ash, carbohydrates, and energy. Following this interaction, the changes found in the energy values are not significant, and thus, it can be concluded that the different preservatives did not show changes in the energy values, which were quite constant, around 397 Kcal. For moisture and proteins, the preservatives seemed to show some significant changes, in which the highest percentage of moisture was found for the preparation preserved with chestnut extract, followed by the chestnut flower and control sample in exequo, which also showed higher values than the sodium benzoate preserved preparation, citric acid and quercetin, all with significant differences among each other. The fact that the chestnut extract preserved nutraceutical showed higher values of moisture was expected, due to the preservative being added in an aqueous form. Furthermore, the chestnut flower, although being dried also showed a higher moisture value than the pure compounds. Still, even though variations in moisture were significative, they ranged between 67% to 70%, which is a very acceptable amount of variation. Interestingly, for the protein quantity, which also showed significative differences among the different nutraceutical preparations, the highest value was found for sodium benzoate preserved formulation, followed by citric acid, quercetin and the nutraceutical with chestnut extract, all in exequo. Sodium benzoate showed the best preserving effect in terms of the protein content. Once again, although there were significative differences between the protein content of the different formulations, the overall amount of protein was very low, under 1 g/100 g, and small changes in amounts had significant differences. Interestingly, over the course of the 45 days hardly any changes occurred in terms of nutritional value and antioxidant capacity. 

### 3.2. Fatty Acids

The lipid fraction analyzed, consisted on the individual fatty acids, which were detected through GC with prior transesterification in methanol, sulphuric acid, and toluene. Table 2 shows the different individual fatty acids found in the different nutraceutical preparations. Although other fatty acids were detected, due to the extremely low amounts, only the 9 most abundant are tabled, namely decanoic acid (C10:0), undecanoic acid (C11:0), lauric acid (C12:0), myristic acid (C14:0), pentadecanoic acid (C15:0), palmitic acid (C16:0), stearic acid (C18:0), oleic acid (C18:1n9c), and decosanoic acid (C22:0). Fatty acids that were detected, but not tabled due to low amount were C8:0, C17:0, C18:n9t, C18:2n6c, C18:3n3, C20:0, C22:2, and C24:0. Although the total fat in the nutraceutical preparations did not go over 0.06 g/100 g, some individual fatty acids showed a relatively high amount, namely palmitic acid which was the most abundant individual fatty acid, followed by stearic acid. Overall, considering the types of fatty acids and the extremely low amount of fat in the nutraceutical preparations, saturated fatty acids (SFA) were the most prevalent, with 88% of the total, followed by monounsaturated fatty acids (MUFA) at 5% to 7% and finally polyunsaturated fatty acids (PUFA) at 3% to 4%. The high amount of saturated fatty acids should not be regarded as a disadvantage of the nutraceutical formulation, given the bad effects of these molecules to human health. Due to the very low fat in the nutraceutical formulation, which is below 0.1 g/100 g, the consumption of this nutraceutical does not pose a threat in terms of SFA. In terms of classification, all samples showed a significative interaction (TP×ST < 0.05), and thus no classifications could be carried out, which further proves that during the 45 days there are hardly any changes in the fatty acid profile. This lack of oxidation could be due to the antioxidant environment created by the pulp of the *A. arborescens* leaves and further potentiated by the preservatives added, which reduced lipid peroxidation.

### 3.3. Chemical Profile

Represented on Table 3 are three different classes of compounds, namely mineral fraction, free sugars, and organic acids. Regarding minerals (left section, Table 3), which were analyzed through atomic absorption spectroscopy (AAS), both micro and macro elements were detected. The microelements consisted of Fe, Cu, Zn, and Mn, while macroelements included Ca, Mg, Na, and K. Overall, the highest mineral element was calcium, followed by sodium, potassium, and magnesium. For all minerals, a significative interaction was found for both factors, ST and TP, and thus no statistical classification was possible, which, considering the elemental nature of these minerals, a significant change was not expected. Still, important conclusions were, especially in terms of the recommended daily intake of these elements. Regarding potassium, it was found between 50 to 64 mg/100 g of fresh weight. Considering that the recommended maximum daily consumption of the nutraceutical formulation is set at 50 g/day (roughly 45 mL), the amount of potassium ingested is far from the daily limit of 3500 mg/day, recommended by EFSA [25]. Sodium, the second highest mineral in all formulations, was detected at 100 mg/100 g, quite low, considering an adequate limit of 1.5 g/day. Sodium, being an important element for the physiological workings of the human body is also responsible for high blood pressure when consumed in excess. Thus, the daily dose of the nutraceutical formulation does not constitute a risk of overconsumption [25,26]. Calcium, the most abundant mineral in the formulations was detected under the daily limit of 2500 mg/day, whilst magnesium did not go over 0.27 mg/100 g of a 350 mg limit. Regarding microelements, manganese and iron where the most abundant, although they did not go over 0.27 and 0.2 mg/100 g, respectively. Considering the highest daily dose of the nutraceutical, the intake of manganese is below the 3 mg/day limit [25,27]. Iron’s daily intake limit is set at 8 to 10 mg/day, being the amount administered by the nutraceutical (45 mL) equivalent to 0.1 mg, and thus, not exceeding the recommended values [25]. Zinc, followed by copper were the least abundant microelements, and as all other minerals, the amounts were very far from the daily intake limit of 9.5 mg/day (7 mg/day woman) for zinc and 5 mg for copper. Overall, the nutraceutical does not pose a threat of overconsumption of the different mineral elements [25]. The fact that metals like Zn and Fe were detected proves the effectiveness of quercetin and chestnut flower extract as preservatives, probably due to one of their antioxidant mechanisms being related to metal chelating capacity, shown in Table 1 for the TBARS assay where they showed a much higher antioxidant activity compared to the other tested preservatives.

The center of Table 3 shows the three detected soluble sugars, namely fructose, glucose, and trehalose. A significant interaction was found for glucose and trehalose, and thus no general conclusions were extracted, likewise for total sugars. Regarding the different formulations, only the nutraceutical preserved with quercetin showed a significatively higher value from the rest of the formulations, showing a higher protection of this simple sugar. Considering ST, fructose showed a significant reduction from T0 to T30, but did not show this tendency from T30 to T45.

Regarding organic acids, (right side, Table 3), only two were detected, namely malic and citric acids, of which malic showed the highest values, maximizing at 0.37 g/100 g, while citric acid did not go over 0.18 g/100 g. Once again, both ST and TP showed interaction, not allowing for any concrete conclusions.

### 3.4. Phenolic Compounds

Data regarding the quantification of the major phenolic compounds, as well as the different families of compounds found in the different nutraceutical formulations are present in Table 4. As Supplementary Material (Table S1), the chromatographic data (retention time, UV-maximum absorption, molecular ion, and fragmentation pattern in MS^2^) of all the phenolic compounds tentatively identified in the different formulations are described. Previously Añibarro-Ortega et al. (2019) performed an in-depth characterization of the phenolic compounds profile of *Aloe vera* leaf, which allowed the tentative identification of peaks 1 (cis 4-O-p-coumaroylquinic acid), 2 (trans 4-O-p-coumaroylquinic acid), 4 (apigenin-6,8-C-diglucoside), 5 (apigenin-C-hexoside-C-hexoside), 9 (aloin B, isobarbaloin), 10 (aloin A, barbaloin), and 11 (2’-p-methoxycoumaroyl aloesin) [28]. Peaks 6 and 12, aloenin and 7-methylether of 2’-feruloylaloesin malonyl-4,5-O-dicaffeoylquinic acid, respectively, were also previously described in Aloe species by El Sayed et al. (2016) [29]. Peaks 3 (2’’-O-pentoside-8-C-hexoside-luteolin), 7 (apigenin-2’’-O-rhamnose-C-hexoside), and 8 (4-(4’-Hydroxyphenyl)-2-butanone-4’-O-β-D-(2’’-O-cinnamoyl-6’’-O-galloyl)glucopyranoside) are, as far as the authors knowledge, firstly described here for Aloe species, having been previously described in other plants by Paucar-Menacho et al. (2017), Roriz et al. (2014) and Jin et al. (2007) [30,31,32].

Although very low amounts of phenolic compounds were found in all nutraceutical preparations, the most abundant were the aloins family and apigenin-6,8-C-diglucoside (peak 4). Despite of a significant interaction among ST and TP, some general conclusions were extracted from the EMM plots for aloenin, total flavonoids, and total non-anthraquinones. The EMM plots are provided as Supplementary Material (Figure S1), where the three time periods are showed in different colors. Section (A) reveals that aloenin reduced along the shelf life for all formulations, although the formulation preserved with chestnut flower showed the highest amounts at T0, and continued to be the highest throughout the whole analysis. Inversely, the control sample showed a lower amount of this polyphenol during the whole course of the experiment. Section B shows the EMM plot of total flavonoids, in which the pattern is very similar, showing a reduction over time, with the control sample showing the lowest amount. The nutraceutical formulation preserved with sodium benzoate and citric acid was the second worst, except for T0 where sodium benzoate showed a higher preservation capacity than citric acid and quercetin. Overall, the nutraceutical preserved with chestnut flower showed the highest quantity of flavonoids, followed by the nutraceutical preserved with the extract of chestnut flower. Finally, Section (C) shows the total phenolic compounds other than anthraquinones, which display a similar behavior to the other plots, namely a reduction over storage time. For T0, the control sample showed similar values to the ones for the formulation with citric acid and quercetin, but, at T30 and T45, the control sample did not preserve the phenolic compounds and thus their quantities were close to zero. Sodium benzoate and citric acid did not preserve the non-anthraquinones as the dry milled chestnut flowers, which was once again the best preservative for phenolic compounds while sodium benzoate showed the least preserving effect of all.

### 3.5. Linear Discriminant Analysis (LDA)

**Figure 1:**
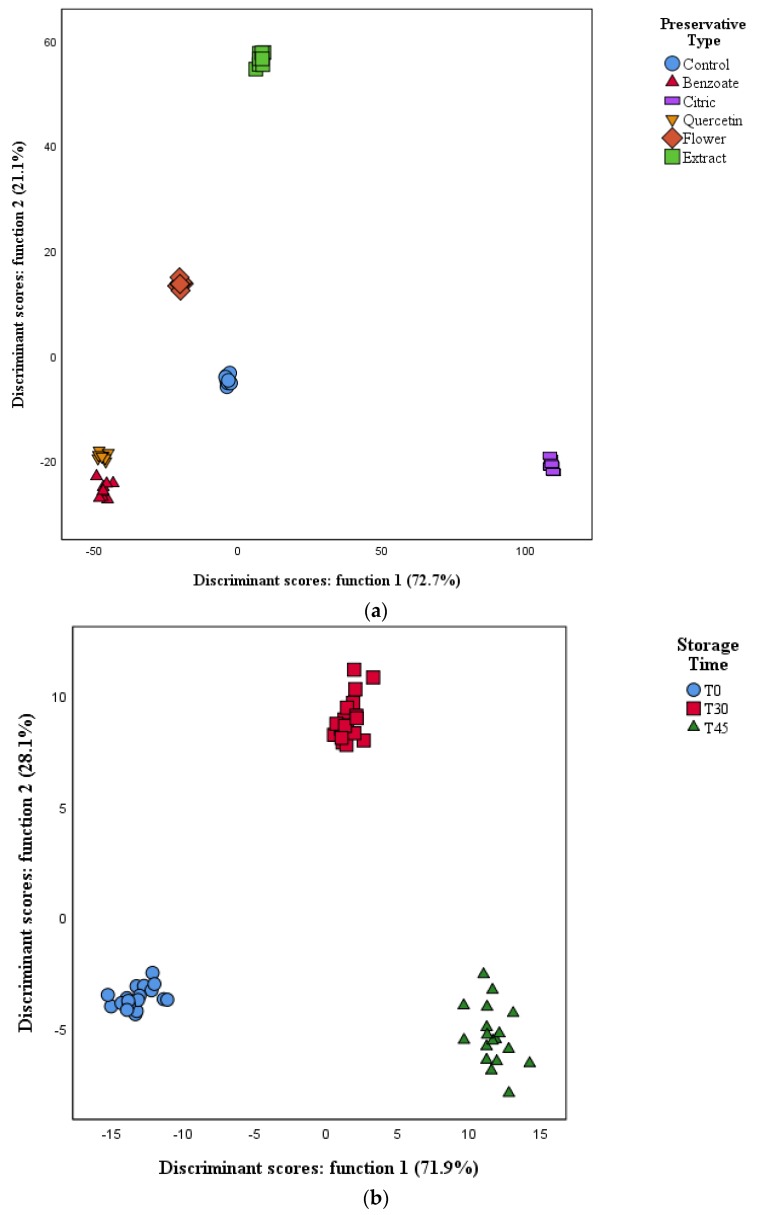
Spatial distribution of TP (**a**) and ST (**b**) markers following the distribution set by the canonical discriminant functions coefficients. Function 1 accounted for 71.9% of the variation, while function 2 accounted for 28.1%. Together, both functions covered 100% of the variation.

Despite the differences found in the formulations preserved with different compounds and extracts, a LDA was used to assess the magnitude of these differences, and if they were enough to discriminate each preservative and storage time, and, if possible, finding the variables that most contributed to this discrimination. Thus, regarding TP (type of preservative), the model defined 5 functions that included 100% of the variance (function 1: 72.7%; function 2: 21.1%; function 3: 5.0%; function 4: 0.8% and function 5: 0.4%) (Figure 1a). Among the 47 analyzed parameters (variables), 18 showed discriminant ability, namely moisture, citric acid, TBARS, total organic acids, proteins, apigenin rhamnose hexoside, manganese, sodium, calcium, SFA, potassium, fructose, zinc, magnesium, fat, energy, glucose, and C22:0 (behenic acid). Of these, the ones mostly correlated with function 1 were magnesium, citric acid and TBARS, while magnesium, C22:0 and manganese were the most correlated with function 2. Function 1 clearly separated the nutraceutical preserved with citric acid from the other preservatives, showing a distinct output, which is especially relevant due to this function accounting for 72.7% of variability. Considering function 2, which separated the remaining samples among each other, it is clear that the sample preserved with the extract of chestnut flower was the most distinct from the others, being the sample preserved with quercetin similar to the positive control (preserved with sodium benzoate). The control sample was equidistant from the benzoate and quercetin preserved samples, and the ones preserved with chestnut flowers.

Figure 1b, pertaining to the ST, the model defined 2 functions that included 100% of the variability, with function 1 accounting for 71.9% and function 2 for 28.1%. Of the 47 analyzed parameters, 8 showed discriminant ability, namely aloenin, aloin B, calcium, C11:0 (undecylic acid), malic acid, OxHLIA60, C10:0 (decanoic acid) and trehalose. The parameters mostly correlated with function 1 were C10:0, C11:0 and malic acid, while C12:0 (dodecanoic acid), OxLHIA60 and malic acid where most correlated with function 2. Interestingly, the most correlated parameters to both functions were fatty acids, which are among the most sensitive molecules to oxidation through lipid peroxidation, making it logical that they would correlate with functions relating to the different storage times. Function 1, accounting for 71.9% of variation, clearly separated T0 from T45, making it understandable due to the changes suffered by all molecules along the 45 days of storage. Function 2 separated T30 from the other two periods, although not being extremely different, provided that function 2 only accounts for 28.1%. Overall, the models allowed to clearly separate the samples by preservative, but also in the different storage periods. The LDA performance was 100% accurate both for the original grouped cases and the cross-validated ones.

### 3.6. Microbiological Analysis

**Figure 2:**
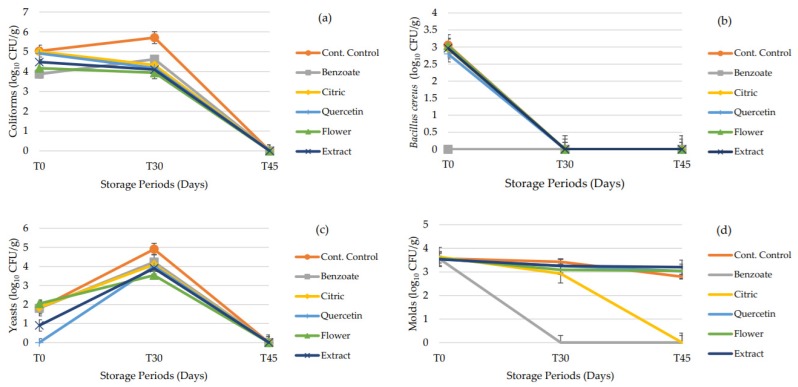
Microbial development along the 45 days of storage time for (**a**) coliforms, (**b**) *Bacillus cereus*, (**c**) yeasts, and (**d**) molds.

The antimicrobial capacity of a food preservative is of paramount importance due to much spoilage of foods being of microbial origin. Considering this, aliquots of the different nutraceutical formulations with the different preservatives were contaminated with various microorganisms immediately after preparation, in order to verify their capacity to delay microbial growth or even eliminate them. A control sample, with no contamination was used as a comparison. Four microbiological analysis were performed with different microorganisms or groups of microorganisms, namely coliforms, *Bacillus cereus*, yeasts and fungi (molds). Regarding the coliforms, Figure 2a, at T0, all formulations show the same load of coliforms. Then, at T30, the contaminated control sample increased the load, while all other formulations reduced this load. Finally, at T45, all formulations showed no load of coliforms, revealing that the substrate for these microorganisms was completely consumed, or the antimicrobial capacity of the *A. arborescens* was enough to eliminate these bacteria. Of all the tested nutraceuticals, the one preserved with sodium benzoate seems to be the least effective on reducing the microbial load, while the one preserved with chestnut flower was the best. Figure 2b shows the count of *Bacillus cereus*, which is a sporulated bacterium known for its food poisoning capabilities and its resistance to thermal treatments. Interestingly, after 30 days, the bacterial load was reduced to zero, from the 3 CFU/g at T0. This reduction could be due to either the substrate of the bacteria being consumed, or once again the antimicrobial capacity of *A. arborescens*, as was verified with *Bacillus cereus*. Regarding yeasts, Figure 2c, these microorganisms showed an increase from T0 to T30 followed by a decrease from T30 to T45. Although all formulations followed this tendency, as expected, the contaminated control showed the highest value at T30, followed by the formulation preserved with sodium benzoate, being the one preserved with chestnut flower the one with the lowest CFU, displaying a better preserving action than the positive control. Finally, regarding molds, in Figure 2d, at T0, immediately after contamination, all formulations showed 3.5 CFU/g which, at T30 for the benzoate preserved samples (positive control), the microbial load was reduced to zero, while being roughly even for all other samples. At T45 the samples preserved with citric acid decreased their microbial load to zero, with all other samples unchanged. In this case, the positive control was the most effective at controlling these contaminants, followed by citric acid preserved formulations. When comparing the microbial count, it should be expected that at T0 the contaminated samples showed higher CFU/g than at T30, but, in the present study the opposite was observed. This can be due to the immediate analysis of the formulations after the contamination procedure giving no time for the microorganisms to adapt to the formulation. By analyzing the microbial results, it can also be seen that in all the formulations contaminated with bacterial strains, the control sample showed the same behavior as the formulations with preservatives, a fact that can be attributed to the acidic pH of the nutraceutical formulation, about 4. Concerning the molds, the load over time was maintained and the low pH did not significantly affect the viability of *A. parasiticus*. Nevertheless, sodium benzoate and citric acid were able to decrease the count of these molds. The same was verified in a study conducted by Battey et al. (2001) that reported the resistance of molds to acidic media and the efficiency of potassium sorbate as a growth inhibitory agent for molds [33]. Regarding the chestnut flower and the citric acid, the most promising agents in this study, their efficacy as antimicrobial agents has already been described in literature, and this work highlights the antimicrobial potential of these ingredients as valuable preservative candidates [34,35,36].

Overall, except for the molds, the chestnut flower seemed to be a suitable antimicrobial preservative for this nutraceutical, being at times better than the commercially used sodium benzoate.

## 4. Conclusions

Choosing a preservative for a food, food supplement or nutraceutical is a tough task, especially if the main added value of the product is the natural origin of its ingredients. Having a naturally produced food or nutraceutical preserved with synthetic additives can be a setback in terms of consumer perception and marketing. The quest for new preservatives must rely on extensive trials and conform with the legislation set by governing bodies. Still, natural preservatives are very well accepted by consumers due to their perception of a more natural food, without synthetic molecules, as well as significative contribution to clean label foods. Thus, in this study, four natural putative preservatives were trialed, and, of these, the ones that stood out as the most promising were the citric acid and dry milled chestnut flower, by showing the best results in controlling the microbial load over the shelf life period and by protecting the phenolic compounds. This exceptional ability to preserve, in some parameters rivalling with sodium benzoate, was further confirmed by the separation achieved in the LDA. Further studies are still needed, namely, to understand their stability during the unopened shelf-life of the nutraceutical preparation. The dry milled chestnut flowers and quercetin should be further studied to then request approval from regulatory instances, and constitute an alternative to synthetic preservatives for nutraceuticals.

## Figures and Tables

**Table 1:** Antioxidant and nutritional profiles of the different nutraceutical formulations. The antioxidant analysis (left of the vertical dashed line) are expressed in mg and µg/mL of extract, respectively, while the nutritional value (right of dashed line) is expressed in fresh weight.

		TBARS	OxHLIA Δ*t* 60 min	OxHLIA Δ*t* 120 min	Moisture (%)	Fat (g/100 g)	Proteins (g/100 g)	Ash (g/100 g)	Carbohydrates (g/100 g)	Energy (Kcal)	Energy (KJ)
TP	Control	4.3 ± 0.5 ^c^	332 ± 192	715 ± 419	67.92 ± 0.05 ^d^	0.06 ± 0.03	0.37 ± 0.01 ^a, b^	0.63 ± 0.02	98.94 ± 0.04	397.8 ± 0.2	1664.3 ± 0.8
	Sodium Benzoate	6.1 ± 0.9 ^e^	273 ± 40	576 ± 161	67.29 ± 0.06 ^c^	0.05 ± 0.02	0.42 ± 0.01 ^d^	0.68 ± 0.02	98.84 ± 0.03	397.8 ± 0.2	1664 ± 3
	Citric Acid	5 ± 2 ^d^	381 ± 101	792 ± 286	67.1 ± 0.1 ^b^	0.05 ± 0.01	0.38 ± 0.01 ^c^	0.62 ± 0.02	98.95 ± 0.03	397.8 ± 0.2	1664 ± 3
	Quercetin	0.037 ± 0.008 ^a^	139 ± 62	208 ± 99	66.33 ± 0.09 ^a^	0.04 ± 0.01	0.378 ± 0.009 ^b, c^	0.61 ± 0.01	98.97 ± 0.03	397.77 ± 0.06	1664.3 ± 0.8
	Chestnut Flower	0.109 ± 0.005 ^a^	228 ± 136	393 ± 244	68.012 ± 0.07 ^d^	0.06 ± 0.02	0.362 ± 0.008 ^a^	0.60 ± 0.03	98.97 ± 0.03	397.8 ± 0.2	1664.3 ± 0.8
	Chestnut Extract	0.6 ± 0.2 ^b^	370 ± 144	962 ± 377	71.05 ± 0.0 4^e^	0.04 ± 0.02	0.39 ± 0.01 ^c^	0.557 ± 0.008	99.01 ± 0.01	397.7 ± 0.3	1664 ± 1
*p*-value (*n* = 5)	Tukey’s HSD text	<0.001	0.009	<0.001	<0.001	0.016	<0.001	<0.001	<0.001	<0.001	<0.001
ST	T0	2 ± 2	184 ± 66	421 ± 207	68 ± 1	0.05 ± 0.01	0.38 ± 0.02	0.61 ± 0.04	98.95 ± 0.06	397.8 ± 0.1	1664 ± 2
	T30	3 ± 3	380 ± 167	881 ± 480	68 ± 1	0.07 ± 0.03	0.39 ± 0.02	0.61 ± 0.04	98.93 ± 0.07	397.9 ± 0.3	1664 ± 1
	T45	3 ± 3	297 ± 119	522 ± 198	68 ± 1	0.039 ± 0.008	0.38 ± 0.02	0.61 ± 0.04	98.96 ± 0.05	397.7 ± 0.1	1664 ± 2
*p*-value (*n* = 15)	Tukey’s HSD test	0.358	<0.001	<0.001	0.954	<0.001	0.347	0.869	<0.001	0.210	0.210
TP×ST (*n* = 90)	*p*-value	0.121	<0.001	<0.001	0.954	0.017	0.169	<0.001	0.001	0.047	0.047

In each row, for the type of preservative (TP) and shelf-life time (ST), different letters mean statistical differences among the preservative type or shelf-life time, with an overall significance level of 0.05. The presented standard deviations were calculated from results obtained under different operational conditions, and, should therefore not be regarded as a measure of precision, rather as the range of the recorded values. TBARS: thiobarbituric acid reactive substances; OxHLIA: oxidative hemolysis inhibition assay; HSD: honest significant difference.

**Table 2:** Fatty acid profile of the different nutraceutical formulations, analyzed through gas chromatography, coupled to a flame ionization detector (GC-FID), expressed in relative percentage.

		C10:0	C11:0	C12:0	C14:0	C15:0	C16:0	C18:0	C18:1n9c	C22:0	SFA	MUFA	PUFA
TP	Control	4.1 ± 0.5	6 ± 2	2.5 ± 0.6	5 ± 1	2.2 ± 0.7	38 ± 4	20 ± 4	6.3 ± 0.9	5 ± 1	88 ± 2	7 ± 1	4.1 ± 0.9
	Sodium Benzoate	5.5 ± 0.8	8 ± 2	2.8 ± 0.5	5.8 ± 0.6	2.0 ± 0.7	34 ± 1	19 ± 4	5.9 ± 0.9	5 ± 1	88 ± 2	7.5 ± 0.8	4 ± 1
	Citric Acid	4 ± 1	5.3 ± 0.7	2.1 ± 0.5	5.8 ± 0.7	2.5 ± 0.4	39 ± 4	19 ± 4	5.8 ± 0.4	5 ± 2	89.9 ± 0.9	6.8 ± 0.3	3.1 ± 0.7
	Quercetin	4 ± 1	6 ± 3	2.3 ± 0.3	5.4 ± 0.3	2.1 ± 0.3	38 ± 5	19 ± 4	5.5 ± 0.7	7 ± 2	89.8 ± 0.6	6.7 ± 0.9	3.4 ± 0.3
	Chestnut Flower	4.3 ± 0.5	5.1 ± 0.9	1.8 ± 0.2	4.2 ± 0.7	1.5 ± 0.2	41 ± 3	20 ± 4	4.2 ± 0.6	6 ± 2	91.0 ± 0.9	5.3 ± 0.7	3.7 ± 0.2
	Chestnut Extract	4.8 ± 0.5	7 ± 1	2.0 ± 0.4	5.3 ± 0.9	1.7 ± 0.3	39 ± 5	18 ± 3	5.3 ± 0.4	6 ± 1	89.9 ± 0.3	6.7 ± 0.2	3.4 ± 0.2
*p*-value (*n* = 5)	Tukey’s HSD text	<0.001	<0.001	<0.001	<0.001	<0.001	<0.001	<0.001	<0.001	0.001	<0.001	<0.001	<0.001
ST	T0	4.3 ± 0.9	6 ± 1	2.1 ± 0.2	5.5 ± 0.4	1.9 ± 0.5	38 ± 2	22 ± 2	5.6 ± 0.9	4.2 ± 0.8	89 ± 1	7.0 ± 0.9	4.0 ± 0.8
	T30	4.1 ± 0.8	5 ± 1	2.1 ± 0.3	5.1 ± 0.6	1.7 ± 0.3	40 ± 4	21 ± 2	5 ± 1	5 ± 2	90 ± 2	6 ± 1	3.9 ± 0.7
	T45	5± 1	7 ± 2	2.5 ± 0.8	5 ± 1	2.5 ± 0.5	37 ± 5	15.6 ± 0.8	5.7 ± 0.6	7 ± 2	90.0 ± 0.9	6.9 ± 0.8	3.1 ± 0.5
*p*-value (*n* = 15)	Tukey’s HSD test	<0.001	<0.001	<0.001	<0.001	<0.001	<0.001	<0.001	0.124	<0.001	<0.001	<0.001	<0.001
TP×ST (*n* = 90)	*p*-value	<0.001	<0.001	<0.001	<0.001	<0.001	<0.001	<0.001	<0.001	<0.001	<0.001	<0.001	<0.001

In each row, for the type of preservative (TP) and shelf-life time (ST). The presented standard deviations were calculated from results obtained under different operational conditions, and, should therefore not be regarded as a measure of precision, rather as the range of the recorded values. SFA: saturated fatty acids; MUFA: monounsaturated fatty acids; PUFA: polyunsaturated fatty acids.

**Table 3:** Mineral profile detected through Atomic Absorption Spectroscopy, presented in mg/100 g. Soluble sugars and organic acids detected through HPLC-RI and HPLC-DAD, respectively, of the different formulations, expressed in g/100 g of fresh weight.

		Potassium	Sodium	Calcium	Magnesium	Manganese	Zinc	Iron	Copper	Fructose	Glucose	Trehalose	Total Sugars	Malic Acid	Citric Acid	Total Organic Acids
Type of Preservative (TP)	Control	57 ± 5	71 ± 8	112 ± 3	37 ± 1	0.25 ± 0.01	0.15 ± 0.01	0.2 ± 0.3	0.05 ± 0.01	13.1 ± 0.7 ^a^	10.8 ± 0.6	0.23 ± 0.03	24 ±1	0.35 ± 0.03	0.03 ± 0.01	0.38 ± 0.04
	Sodium Benzoate	56 ± 2	70 ± 6	105 ± 3	34.1 ± 0.8	0.23 ± 0.01	0.15 ± 0.01	0.14 ± 0.08	0.06 ± 0.01	12.6 ± 0.7 ^a^	10.5 ± 0.9	0.23 ± 0.05	23 ± 2	0.31 ± 0.03	0.02 ± 0.01	0.34 ± 0.04
	Citric Acid	57 ± 4	66 ± 3	109 ± 9	33 ± 1	0.25 ± 0.01	0.14 ± 0.01	0.15 ± 0.05	0.05 ± 0.01	13.3 ± 0.6 ^a^	11.0 ± 0.4	0.25 ± 0.05	24 ± 1	0.35 ± 0.06	0.18 ± 0.04	0.5 ± 0.1
	Quercetin	64 ± 6	58 ± 7	101 ± 13	33 ± 2	0.23 ± 0.01	0.14 ± 0.01	0.12 ± 0.09	0.05 ± 0.01	14.5 ± 0.9 ^b^	11.6 ± 0.7	0.31 ± 0.07	26 ± 1	0.32 ± 0.05	0.02 ± 0.01	0.34 ± 0.05
	Chestnut Flower	56 ± 5	61 ± 7	100 ± 15	33 ± 3	0.27 ± 0.02	0.13 ± 0.02	0.09 ± 0.01	0.05 ± 0.01	13 ± 1 ^a^	11 ± 1	0.23 ± 0.07	24 ± 2	0.37 ± 0.04	0.03 ± 0.01	0.40 ± 0.04
	Chestnut Extract	50 ± 4	60 ± 3	97 ± 6	32 ± 2	0.25 ± 0.01	0.12 ± 0.01	0.07 ± 0.01	0.05 ± 0.01	13 ± 1 ^a^	11 ± 1	0.21 ± 0.04	24 ± 2	0.31 ± 001	0.02 ± 0.01	0.33 ± 0.02
*p*-value (n = 5)	Tukey’s HSD text	0.105	<0.001	<0.001	<0.001	<0.001	0.025	<0.001	0.056	0.001	<0.001	0.009	<0.001	<0.001	<0.001	<0.001
Shelf-Life Time (ST)	T0	57 ± 8	67 ± 15	105 ± 12	33 ± 3	0.25 ± 0.01	0.14 ± 0.02	0.2 ± 0.2	0.05 ± 0.01	14.1 ± 0.3 ^b^	11.9 ± 0.6	0.27 ± 0.03	26 ± 1	0.35 ± 0.05	0.06 ± 0.07	0.4 ± 0.1
	T30	56 ± 5	68 ± 16	101 ± 10	33 ± 2	0.24 ± 0.02	0.14 ± 0.01	0.09 ± 0.04	0.05 ± 0.01	12.9 ± 0.3 ^a^	10.5 ± 0.7	0.24 ± 0.05	24 ± 1	0.33 ± 0.03	0.05 ± 0.05	0.37 ± 0.05
	T45	58 ± 4	71 ± 13	107 ± 8	35 ± 2	0.25 ± 0.02	0.14 ± 0.01	0.07 ± 0.01	0.06 ± 0.01	13.2 ± 0.6 ^a^	10.7 ± 0.9	0.23 ± 0.08	24 ± 2	0.33 ± 0.04	0.04 ± 0.05	0.38 ± 0.05
*p*-value (n = 15)	Tukey’s HSD test	<0.001	<0.001	<0.001	<0.001	<0.001	<0.001	<0.001	0.005	<0.001	<0.001	<0.001	<0.001	<0.001	<0.001	<0.001
TP×ST (n = 90)	*p*-value	<0.001	<0.001	<0.001	<0.001	<0.001	<0.001	<0.001	0.005	0.051	<0.001	<0.001	<0.001	<0.001	<0.001	<0.001

In each row, for the type of preservative (TP) and shelf-life time (ST), different letters mean statistical differences among the preservative type or shelf-life time, with an overall significance level of 0.05. The presented standard deviations were calculated from results obtained under different operational conditions, and, should therefore not be regarded as a measure of precision, rather as the range of the recorded values.

**Table 4:** Phenolic compounds tentatively identified in the different formulations, expressed in µg/100 g of fresh weight.

		Apigenin-6,8-C-diglucoside*	Apigenin-2’’-O-rhamnose-C-hexoside*	Aloenin**	Aloin B**	Aloin A**	2’-p-methoxycoumaroyl aloesin**	Total Phenolic Acids	Total Flavonoids	Total Aloin	Total non- Anthraquinones	Total Anthroquinones
TP	Control	0.012 ± 0.003	0.009 ± 0.001	0.08 ± 0.04	0.015 ± 0.003	0.014 ± 0.004	0.004 ± 0.006	0.005 ± 0.001	0.09 ± 0.04	0.03 ± 0.006	0.09 ± 0.04	0.12 ± 0.05
	Sodium Benzoate	0.009 ± 0.007	0.008 ± 0.001	0.11 ± 0.04	0.015 ± 0.001	0.013 ± 0.005	0.003 ± 0.002	0.005 ± 0.001	0.12 ± 0.04	0.03 ± 0.01	0.12 ± 0.04	0.14 ± 0.04
	Citric Acid	0.01 ± 0.01	0.010 ± 0.001	0.10 ± 0.03	0.012 ± 0.01	0.011 ± 0.008	0.001 ± 0.001	0.005 ± 0.001	0.11 ± 0.03	0.02 ± 0.02	0.10 ± 0.03	0.13 ± 0.04
	Quercetin	0.024 ± 0.003	0.02 ± 0.001	0.11 ± 0.02	0.020 ± 0.001	0.019 ± 0.002	0.009 ± 0.003	0.006 ± 0.001	0.13 ± 0.01	0.040 ± 0.001	0.12 ± 0.02	0.16 ± 0.01
	Chestnut Flower	0.029 ± 0.002	0.011 ± 0.001	0.14 ± 0.02	0.015 ± 0.001	0.013 ± 0.006	0.02 ± 0.01	0.008 ± 0.002	0.16 ± 0.02	0.028 ± 0.009	0.17 ± 0.04	0.20 ± 0.04
	Chestnut Extract	0.027 ± 0.004	0.011 ± 0.001	0.13 ± 0.03	0.020 ± 0.001	0.013 ± 0.009	0.01 ± 0.02	0.007 ± 0.001	0.14 ± 0.03	0.03 ± 0.01	0.15 ± 0.05	0.18 ± 0.06
*p*-value (*n* = 5)	Tukey’s HSD text	<0.001	<0.001	<0.001	<0.001	<0.001	<0.001	<0.001	<0.001	<0.001	<0.001	<0.001
ST	T0	0.019 ± 0.005	0.010 ± 0.001	0.15 ± 0.02	0.016 ± 0.001	0.019 ± 0.003	0.02 ± 0.01	0.006 ± 0.002	0.16 ± 0.02	0.036 ± 0.005	0.17 ± 0.03	0.21 ± 0.03
	T30	0.01 ± 0.01	0.012 ± 0.001	0.10 ± 0.02	0.010 ± 0.001	0.009 ± 0.006	0.004 ± 0.004	0.005 ± 0.001	0.1 ± 0.02	0.02 ± 0.01	0.11 ± 0.02	0.13 ± 0.03
	T45	0.024 ± 0.009	0.012 ± 0.004	0.08 ± 0.02	0.019 ± 0.001	0.013 ± 0.004	0.004 ± 0.004	0.006 ± 0.001	0.10 ± 0.03	0.032 ± 0.008	0.09 ± 0.03	0.12 ± 0.03
*p*-value (*n* =15)	Tukey’s HSD test	<0.001	<0.001	<0.001	<0.001	<0.001	<0.001	<0.001	<0.001	<0.001	<0.001	<0.001
TP×ST (*n* = 90)	p-value	<0.001	<0.001	<0.001	<0.001	<0.001	<0.001	<0.001	<0.001	<0.001	<0.001	<0.001

In each row, for the type of preservative (TP) and shelf-life time (ST). The presented standard deviations were calculated from results obtained under different operational conditions, and, should therefore not be regarded as a measure of precision, rather as the range of the recorded values. Standard calibration curves: *apigenin-6-C-glucoside (*y* = 107025*x* + 61531, *R*² = 0.9989, Limit of Detection = 0.19 µg/mL and Limit of Quantification= 0.63 µg/mL) and **aloin A (*y* = 3859.4*x* + 21770, *R*² = 0.9992, LOD = 0.36 µg/mL and LOQ = 0.94 µg/mL).

## Supplementary Materials

The following are available online at https://www.mdpi.com/2076-3921/9/4/281/s1, Figure S1: EMM Plots of aloenin, total flavonoids and total non-anthraquinones, Table S1: Phenolic compounds identification.
