# Supplementary material for: Effect of Natural Preservatives on the Nutritional Profile, Chemical Composition, Bioactivity and Stability of a Nutraceutical Preparation of Aloe arborescens
Source: Antioxidants (Basel). 2020 Mar 26;9(4):281. doi: 10.3390/antiox9040281 (PMC7222173; doi:10.3390/antiox9040281)
Supplement: Supplementary file 1 [file antioxidants-09-00281-s001.pdf]

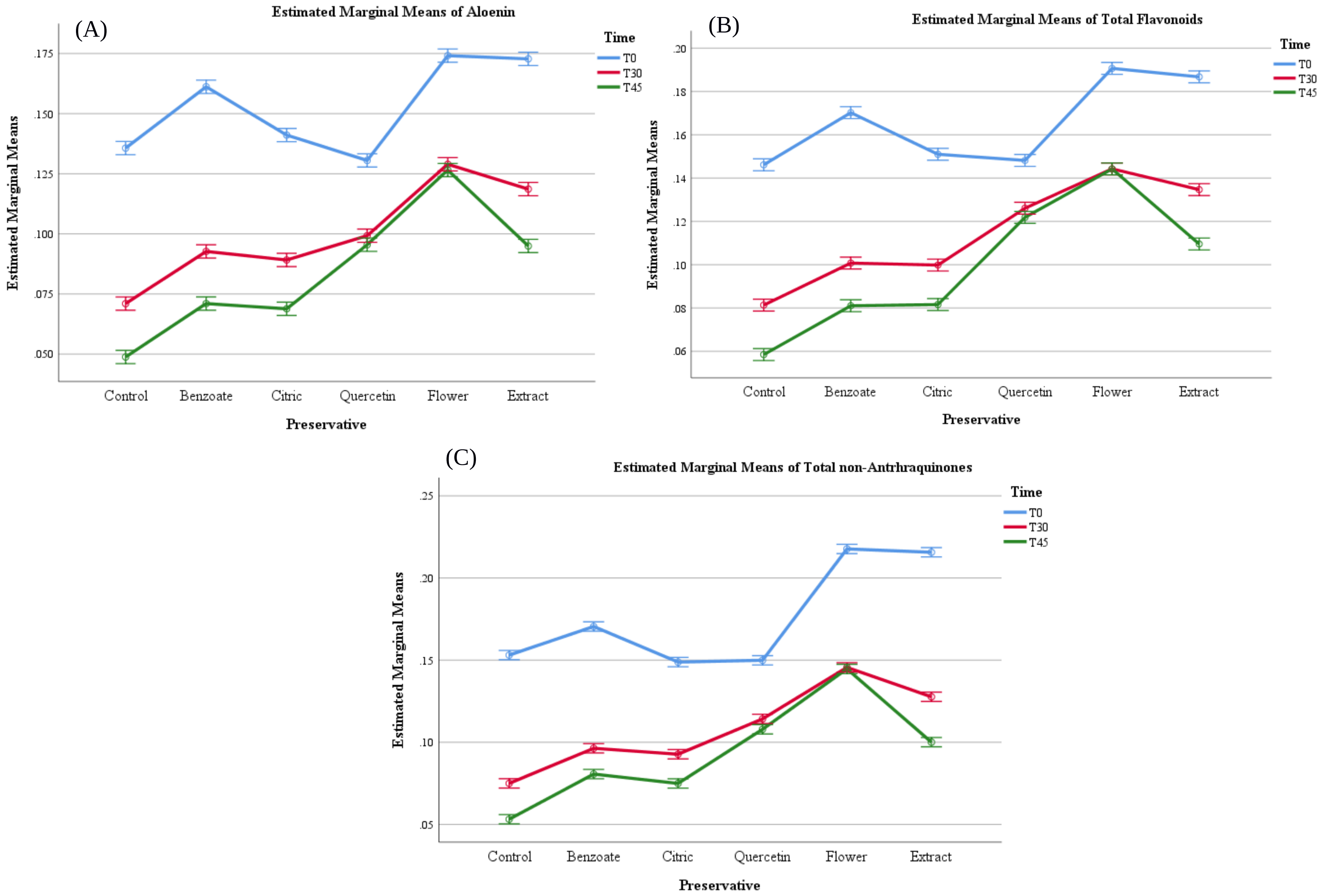

Supplementary Material S1. Estimated marginal means plots for aloenin (A), total flavonoids (B) and total non-anthraquinones (C).

Supplementary Material S2. Retention time (Rt), wavelengths of maximum absorption in the visible region (λmax), mass spectral data, and tentative identification of the phenolic compounds present in the hydroethanolic extracts of of the different nutraceutical formulations.

Peak	Rt (min)	λmax (nm)	$[M-H]^-$ (*m/z*)	MS^2 (*m/z*)	Tentative identification
**1**	11.46	310	337	191(100),173(5),163(11),135(5)	*cis* 4-*O*-*p*-coumaroylquinic acid
**2**	12.55	310	337	191(100),173(5),163(15),135(6)	*trans* 4-*O*-*p*-coumaroylquinic acid
**3**	13.74	343	579	459(85),429(100),357(30),327(3),309(13)	2''-*O*-pentoside-8-C-hexoside-luteolin
**4**	14.66	348	593	473(100),431(14),311(10)	Apigenin-6,8-C-diglucoside
**5**	15.43	344	593	473(100),431(14),311(10)	Apigenin-C-hexoside-C-hexoside
**6**	16.05	297	409	247(100)	Aloenin
**7**	17.56	271/344	577	457(7),413(100),341(5),311(5),293(29)	Apigenin-2''-*O*-rhamnose-C-hexoside
**8**	18.39	271/344	607	443(100), 295(30), 169(80), 125(15)	4-(4'-Hydroxyphenyl)-2-butanone-4'-*O*-β-D-(2''-*O*-cinnamoyl-6''-*O*-galloyl)glucopyranoside
**9**	24.22	220/269/298/354	417	297(100),255(5)	Aloin B (isobarbaloin)
**10**	25.99	220/269/298/354	417	297(100),255(5)	Aloin A (barbaloin)
**11**	27.86	252/301	553	407(100),375(5),347(14)275(47),259(3),233(4),191(2)	2'-*p*-methoxycoumaroyl aloesin
**12**	28.88	244/301	583	407(100),389(30),243(5)	7-methylether of 2'-feruloylaloesin malonyl-4,5-O-dicaffeoylquinic acid
